# The synthesis of active pharmaceutical ingredients (APIs) using continuous flow chemistry

**DOI:** 10.3762/bjoc.11.134

**Published:** 2015-07-17

**Authors:** Marcus Baumann, Ian R Baxendale

**Affiliations:** 1 Department of Chemistry, Durham University, South Road, DH1 3LE Durham, United Kingdom

**Keywords:** continuous processing, flow synthesis, in-line analysis, manufacture, pharmaceuticals, scalability

## Abstract

The implementation of continuous flow processing as a key enabling technology has transformed the way we conduct chemistry and has expanded our synthetic capabilities. As a result many new preparative routes have been designed towards commercially relevant drug compounds achieving more efficient and reproducible manufacture. This review article aims to illustrate the holistic systems approach and diverse applications of flow chemistry to the preparation of pharmaceutically active molecules, demonstrating the value of this strategy towards every aspect ranging from synthesis, in-line analysis and purification to final formulation and tableting. Although this review will primarily concentrate on large scale continuous processing, additional selected syntheses using micro or meso-scaled flow reactors will be exemplified for key transformations and process control. It is hoped that the reader will gain an appreciation of the innovative technology and transformational nature that flow chemistry can leverage to an overall process.

## Introduction

The last 20 years have witnessed a true renaissance in the way synthetic chemistry is performed due to the implementation of various enabling technologies allowing the modern synthesis chemist to select from a range of tools and equipment to best perform a given transformation [1–6]. The trend to question the suitability of classical laboratory glassware and to utilise more ‘fit for purpose’ synthesis equipment not only allows the individual chemists to conduct their research in a more modern fashion, but also adjusts their mind-set towards the full practical breadth of synthesis planning. In this way chemists are more aware of the entire processing sequence, considering quenching, work-up, extraction and purification as part of the holistic design of the preparative route. The introduction of such thinking earlier in a compound’s development pipeline significantly simplifies the scaling transitions required to meet the increasing quantities of material needed for the different stages of biological and regulatory testing and then on into the building of the manufacturing route.

Arguably one of the most widely amenable of the enabling technologies is flow chemistry, which accommodates small foot-print reactors in which streams of substrates and reagents can be united to react in a highly controlled and reproducible environment [7–15]. Importantly, regulation of many parameters such as heat and mass transfer, mixing and residence times are much improved over related batch processes. Advantageously the flow reactor configuration can also be readily customised to meet the specific demands of the reaction and the continuous processing requirements. The construction of the reactor is often modular being assembled from several specialised yet easily integrated components such as heating and cooling zones, micro-mixers, residence tubing coils, separators, and diagnostic/analysis units. This workflow not only allows for facile automation and continuous operation of such processes, but also enables the chemist to perform more potentially hazardous and otherwise forbidden transformations in a safer and more reliable fashion [16–21]. The main advantages cited for improved operational safety are principally the reduced inventories of reactive chemicals, the small contained reactor units and the ability to install real time monitoring of the system leading to rapid identification of problems and the instigation of automated safe shutdown protocols. Furthermore, the use of direct in-line purification and analysis techniques can be implemented thus generating a more streamlined and information enriched reaction sequence [22–26]. Consequently, numerous studies have been published in recent years detailing the beneficial outcome of flow chemistry applied to single or indeed multi-step syntheses of target compounds on various reaction scales [27–34]. At the same time a number of limitations and challenges to the wider adoption of flow chemistry have been identified including reactor fouling, high investment costs and training of the next generation of chemists needed in order to embrace the value of these modern synthesis instruments [35–39].

In order to evaluate the current standing of this field, we will review and discuss several flow based API’s syntheses conducted by scientists from both academia and industry. It is hoped that the reader will through this review gain a greater appreciation of the range of flow chemistries that have already been successfully performed as well as knowledge of some of the more common pitfalls and limitations. Recognition of the problematic aspects of flow chemistry is essential to allow a unified effort from the chemistry and chemical engineering communities in order to surmount these obstacles and for us to achieve the vision of true continuous manufacture of pharmaceuticals.

## Review

### Early flow processing approaches

**Figure 1:**
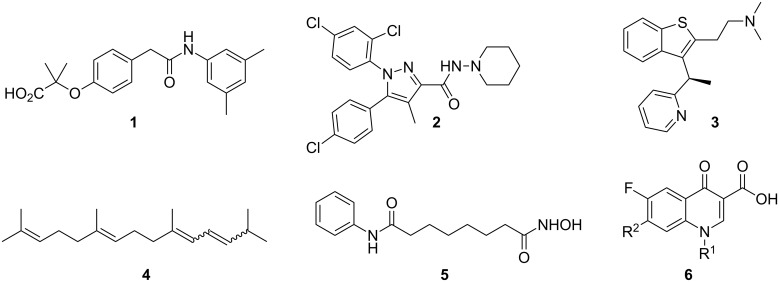
Pharmaceutical structures targeted in early flow syntheses.

The first published examples of flow chemistry applied to the synthesis of pharmaceutically active molecules emerged in the early 2000s when several research groups reported on specific flow transformations that enabled a new synthesis of these known pharmaceuticals. Examples of these early endeavours include the syntheses of efaproxiral (**1**) and rimonabant (**2**) using a AlMe_3_-mediated direct amidation in flow [40], an improved metalation step in the scaled synthesis of NBI-75043 (**3**) [41], a continuous dehydration process to deliver over 5 kg of dehydropristane **4**, a precursor of the immunoactivating agent pristane [42] or the flow synthesis of hydroxamic acids by a procedure that was also applied to the preparation of suberoylanilide hydroxamic acid (**5**, SAHA, Figure 1) [43]. Another early application of microreactor technology was reported in 2005 detailing the assembly and subsequent decoration of the fluoroquinolinone scaffold **6** resulting in the synthesis of a library of analogues including the well-known antibiotic ciprofloxacin (**6**, R^1^ = cyclopropyl, R^2^ = piperazinyl) [44].

**Scheme 1:**
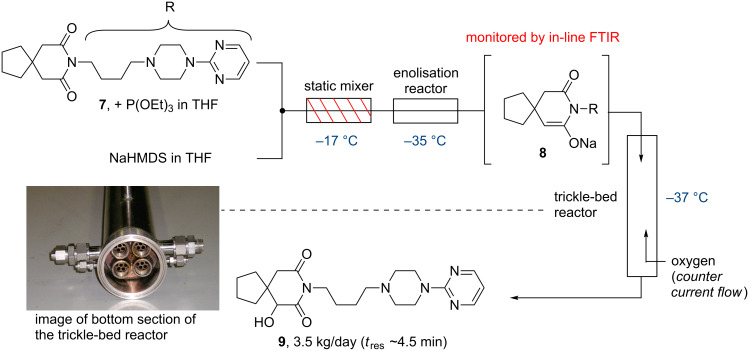
Flow synthesis of 6-hydroxybuspirone (**9**). Inserted photograph reprinted with permission from [45]. Copyright 2008 American Chemical Society.

An important early industry-based example was disclosed by scientists at Bristol-Myers Squibb in 2008 detailing a flow approach towards converting the psychotropic agent buspirone (**7**) into its major active metabolite, 6-hydroxybuspirone (**9**) [45]. This work comprised three consecutive flow steps including a low-temperature enolisation of buspirone (**7**). The subsequent reaction of the enolate with gaseous oxygen in a trickle-bed reactor was coupled to a direct in-line quench of the reaction mixture to yield 6-hydroxybuspirone (Scheme 1).

This approach furthermore made use of in-line analysis techniques like FTIR (for the monitoring of the enolisation step) and was successfully run at steady state for 40 h generating the target compound at multi-kilogram scale. As this paper states, the main advantages of a continuous approach over batch processing in this scale-up campaign were found to be related to safety, isolated purity and economics.

**Figure 2:**
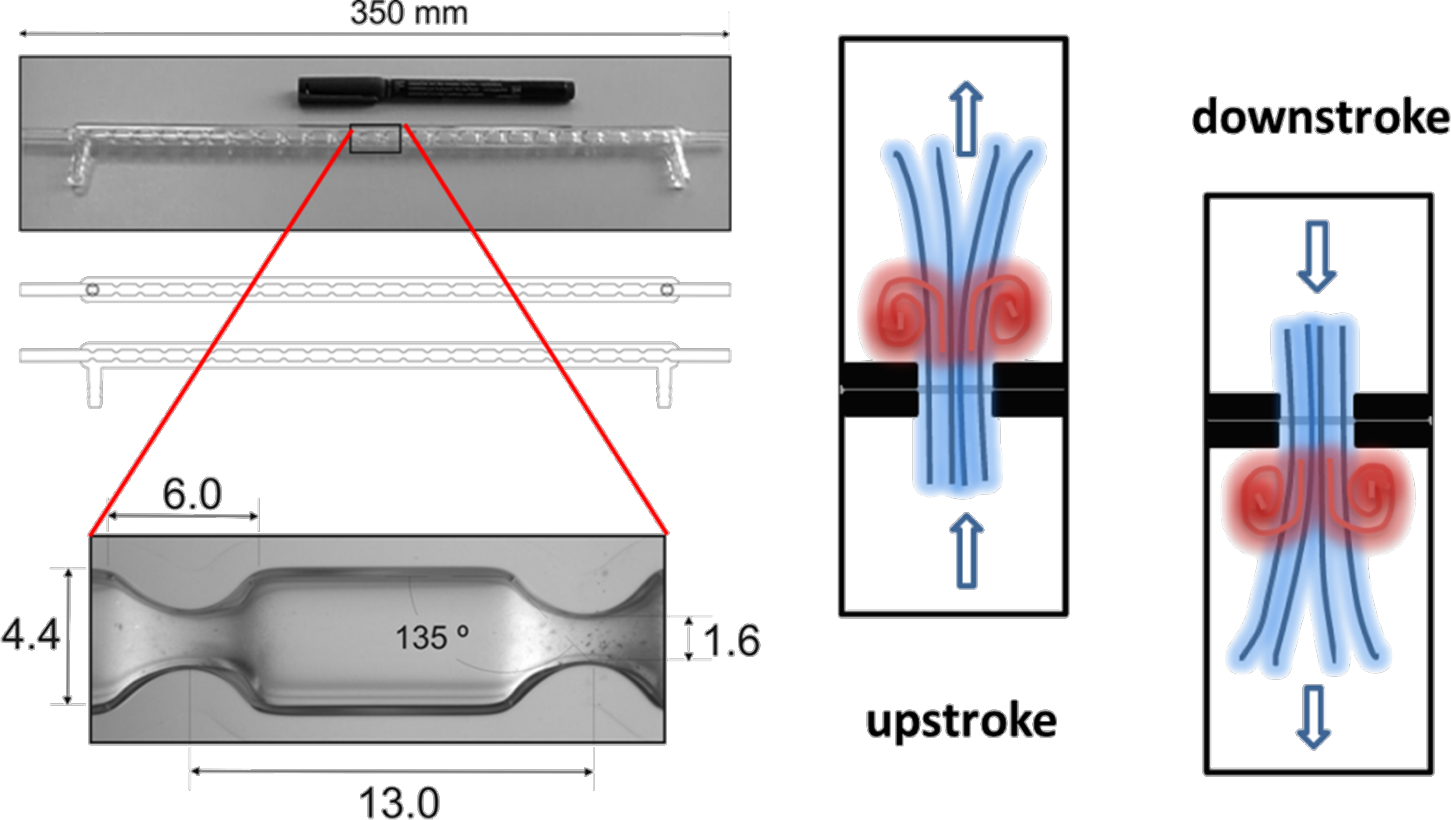
Configuration of a baffled reactor tube (left) and its schematic working principle (right).

The successful outcome of the above study can in part be ascribed to the use of a static mixing device which allowed for the selective and clean mono-deprotonation under scale-up conditions. This was in stark contrast to the related batch scenarios which were difficult to control. Owing to the importance of efficient micro-mixing attainable in continuous processing another interesting reactor design coined as a ‘continuous oscillatory baffled reactor’ (COBR) was introduced. In this set-up the reactor stream being processed is directed into a tubular reactor which contains periodically spaced annular baffles thereby creating a series of eddies through oscillatory motion simultaneously applied to the reactor (Figure 2) [46]. The resulting vigorous axial and radial mixing results in very sharp residence time distributions and excellent heat and mass transfer. Consequently, long batch processes (including crystallisations, fermentations, polymerisations or waste water treatments) can be translated into a continuous process. In an early example such COBRs were applied to the flow synthesis of aspirin showcasing the effectiveness of this reactor type during a week long campaign delivering the target compound at scale with very high product purity (99.94%) and minimal loss of product during cleaning (<0.005%) [47].

**Scheme 2:**
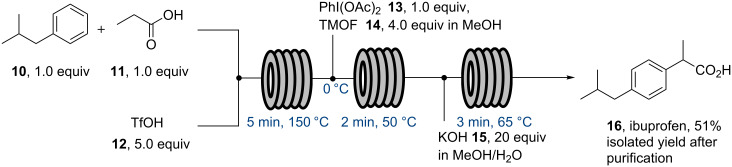
McQuade’s flow synthesis of ibuprofen (**16**).

In 2009 a flow synthesis of a high volume pharmaceutical was reported by the McQuade group describing a three step approach towards ibuprofen (**16**) using microreactor technology [48]. A fully continuous process was aspired to, in which only final purification was to be performed off-line at the end of the sequence. Each of the individual steps were first optimised in flow being mindful of the reagents used in order to avoid downstream incompatibilities. The initial step was a Friedel–Crafts acylation of isobutylbenzene (**10**) with propionic acid (**11**) in the presence of excess triflic acid (**12**). The transformation was found to work very effectively and the acid catalyst was also tolerated in the subsequent 1,2-aryl migration step. This was mediated by a hypervalent iodine reagent, PhI(OAc)_2_ (**13**), conducted in trimethyl orthoformate (**14**, TMOF) and methanol (Scheme 2). The direct saponification of the resulting rearranged methyl ester with an excess of base thus completed the telescoped flow synthesis of ibuprofen (**16**). Work-up, via acidification and repeated washes with ether, water and brine, followed by filtration, evaporation, treatment with active carbon and finally recrystallisation was performed manually to eventually yield pure ibuprofen product (99%). Overall this pioneering work allowed for the synthesis of ibuprofen in only ten minutes residence time albeit in a yield of only 51% equating to a productivity of 9 mg/min.

Although this work nicely demonstrates the feasibility of constructing a continuous process it is mainly an academic proof of principle based upon an important well known molecule. We state this not to detract from the work but to comment here about the different approaches and considerations that generally focus the minds of academics and industrialists and use this example as illustration.

This route would certainly not constitute an economically viable approach compared to the existing manufacturing routes which have been highly refined and optimised [49–51]. Although modern reagents such as hypervalent iodine and triflic acid represent very valuable additions to the chemists’ repertoire they are also inherently expensive and difficult to source at scale. In addition the waste streams generated through their use would also be difficult and costly to dispose. This aptly leads to an interesting relationship that is often encountered in innovative work employing new technologies where a general mind set exists to also test the limits of modern reagent equivalents in addition to the equipment. From an academic perspective this is a positive and beneficial contribution to the progression of the subject, however, this can significantly restrict the translational value of the methodology with respect to adoption or convenient uptake by industry. Commonly industry cites cost, unacceptable solvent combinations and limited availability of new reagents (metal ligand combinations) at scale as the main hindrances to uptake. This message is certainly being acknowledged with many of the more recent publications originating from academia using industry evaluation metrics and reagent selection guides to influence their route selection.

However, it is not only academia which is in the firing line, industry scientists are often heavily criticised as being too reliant on existing reactions/reagents and therefore being too conservative and resistant to change. Although this is often a corporate promoted strategy resulting from being risk adverse it can bias mind sets to fall back on the proven rather than innovate and explore. The additional pressures of meeting regulatory compliance, which is often easier based upon precedent, and the constant ‘time = money’ equation also compound the effect. Again such perceptions are changing with many companies creating specialist innovation groups dedicated to exploration and exploitation of new technologies. Fledgling innovations are in-house tested, monitored and if viable rolled out more expansively throughout the company. An excellent illustration would be the adoption of microwave reactors which have become primary heating methods in many medicinal chemistry labs. This is also being seen in the adoption of flow processing technologies where all the major pharmaceutical companies have internal teams working on business critical projects as well as longer term objectives. Furthermore the generation of various consortia between academia and industry is also influencing the transfer of knowledge, reasoning and importantly expectations. All these considerations are helping to drive the area of flow chemistry.

### Flow processing scenarios

**Scheme 3:**
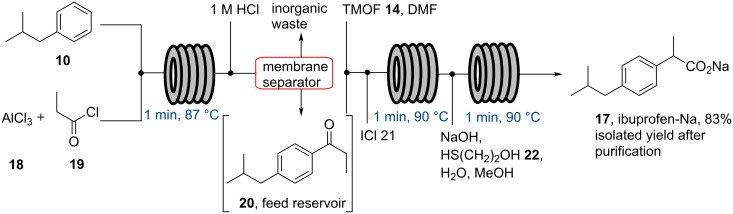
Jamison’s flow synthesis of ibuprofen sodium salt (**17**).

Recently, the Jamison laboratory reported on an improved flow synthesis of the ibuprofen sodium salt (**17**) that delivers the target compound in only three minutes residence time with an improved productivity of about 135 mg/min [52]. As the key steps are the same as in McQuade’s approach (Friedel–Crafts acylation, 1,2-aryl migration and saponification) this report focuses on improved output by intensifying the overall sequence (Scheme 3). As such an in-line extraction is performed after the Friedel–Crafts acylation step, followed by dissolving intermediate **18** in trimethyl orthoformate and DMF. This stream is then combined with a stream of ICl (**21**) to affect the 1,2-aryl migration in a heated flow reactor (1 min, 90 °C) followed by treatment of the stream with NaOH, 2-mercaptoethanol, MeOH and water in order to hydrolyse the intermediate methyl ester and quench residual ICl. After collection of the crude reaction mixture an extractive work-up was performed off-line, in which ibuprofen was generated upon acidification from its sodium salt **17**.

**Scheme 4:**
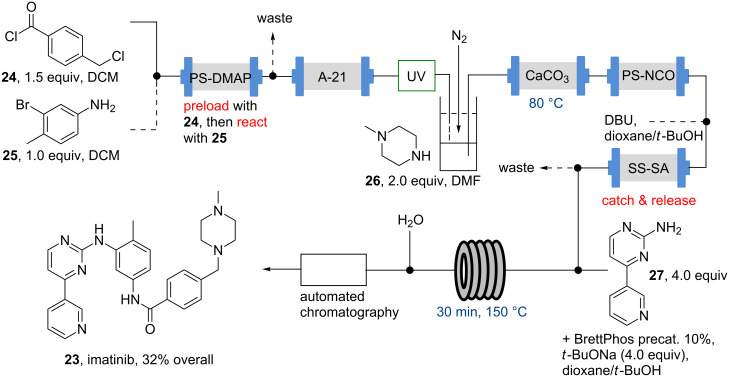
Flow synthesis of imatinib (**23**).

Another high profile pharmaceutical for which a flow synthesis has been developed is imatinib (**23**), the API of Novartis’ tyrosine kinase inhibitor Gleevec [53–55]. Reported by the Innovative Technology Centre (ITC) in 2010, this landmark synthesis was realised as a continuous process featuring an amide formation, a nucleophilic substitution and a Buchwald–Hartwig coupling as key synthesis steps performed in flow (Scheme 4).

Further highlights of this approach were the use of scavenger resins for intermediate purification and solvent switching operations as well as the use of in-line UV-monitoring needed to orchestrate the various reagent streams. Although the low solubility of various intermediates proved challenging, the designed route was able to successfully deliver sufficient quantities of imatinib (**23**) and several of its analogues (~30–50 mg each) in high purity within one working day allowing subsequent testing of new derivatives. Although this approach was conducted as a fully integrated telescoped continuous flow sequence its capacity to run as an uninterrupted process is certainly limited by the solid-phase scavengers employed as purification aids. The stoichiometric scavenging capacity of many of these species coupled with their limited loadings does restrict the quantities of material which can be generated in a run. As a consequence this approach is better suited to the rapid formation of small quantities of directly purified material for screening purposes but does not constitute a viable mode of performing direct large scale manufacture.

**Scheme 5:**
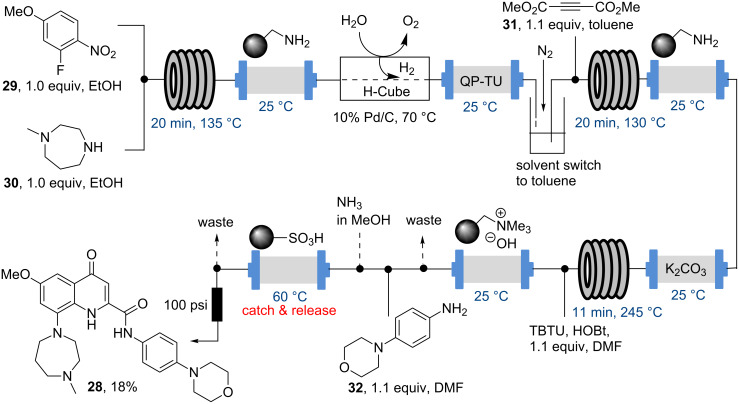
Flow synthesis of the potent 5HT1B antagonist **28**.

In the same year the ITC also reported on their efforts towards the flow syntheses of two lead compounds reported earlier by AstraZeneca. The first one details the flow synthesis of a potent 5HT1B antagonist (**28**) that was assembled through a five step continuous synthesis including a S_N_Ar reaction, heterogeneous hydrogenation, Michael addition–cyclisation and final amide formation (Scheme 5) [56]. This sequence again makes use of in-line scavenging resins for purification purposes and demonstrates the utility of in-line solvent switching protocols and high temperature reactor coils operating at 130–245 °C, well above the boiling points of the solvents employed.

**Scheme 6:**
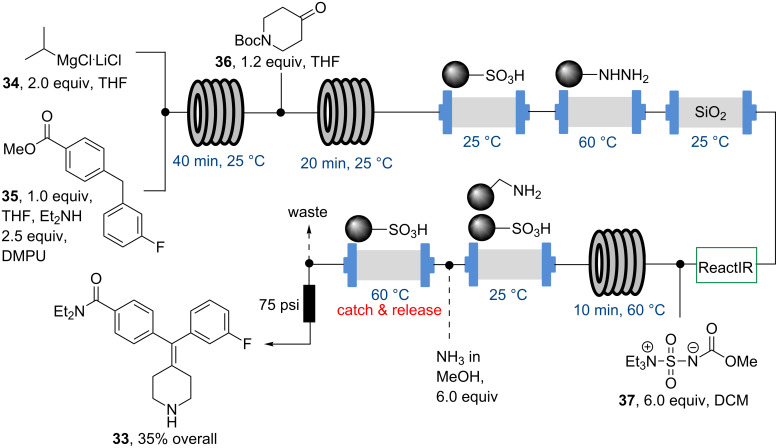
Flow synthesis of a selective δ-opioid receptor agonist **33**.

In the second study, the flow synthesis of the selective δ-opioid receptor agonist **33** was discussed (Scheme 6) [57]. Again, a strategy of integrating each of the three synthetic steps with a sequenced cascade of scavenger agents to perform the aspects of work-up and purification was used.

The report also showcased the generation and use of organometallic species (i.e., Grignard reagents) in flow synthesis as well as in-line React-IR monitoring in order to precisely control the onset of late stage flow streams that are affected by dispersion effects thus marking the first use of this now commonly incorporated analysis technique.

As the safe use of organometallic reagents has emerged as a key facet of flow chemical synthesis [58], the ITC reported on the design and implementation of a dual injection loop system that could deliver solutions of organometallic reagents (i.e., LiHMDS or *n*-BuLi) as a pseudo-continuous process [59]. This protocol enables loading of a second loop with the unstable organometallic reagent whilst the first loop (previously filled with the same solution) is being directed to the intended flow transformation. Once this first reagent loop is empty, an automated protocol switches the valves so that the second loop transfers the reagent, whilst the first one is being recharged.

**Scheme 7:**
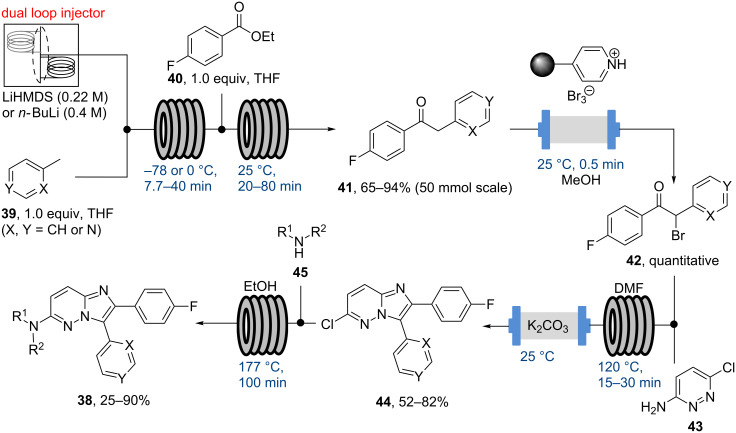
Flow synthesis of a casein kinase I inhibitor library (**38**).

This concept was successfully applied to the flow synthesis of a 20-member library of casein kinase I inhibitors (**38**) that also demonstrate the selective mono-bromination, heterocycle formations and high temperature S_N_Ar reactions as key flow steps in the sequence (Scheme 7).

**Scheme 8:**
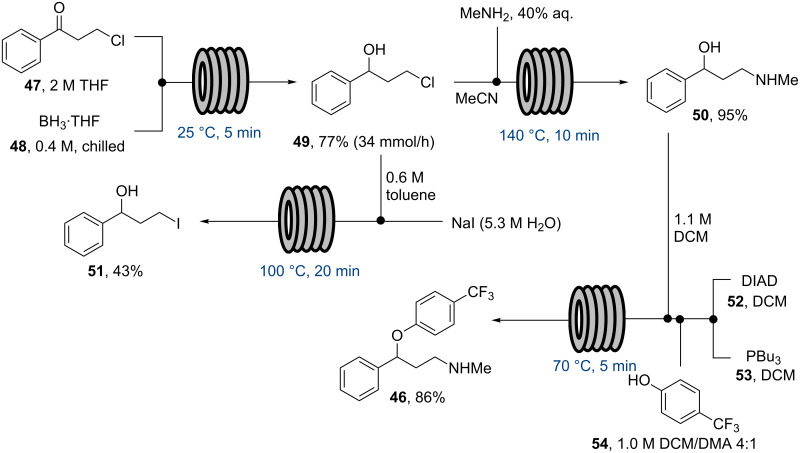
Flow synthesis of fluoxetine (**46**).

One of the early published examples of industry-based research on multi-step flow synthesis of a pharmaceutical was reported in 2011 by scientists from Eli Lilly/UK and detailed the synthesis of fluoxetine **46**, the API of Prozac [60]. In this account each step was performed and optimised individually in flow, with analysis and purification being accomplished off-line. The synthesis commences with the reduction of the advanced intermediate ketone **47** using a solution of pre-chilled borane–THF complex (**48**) to yield alcohol **49** (Scheme 8). Conversion of the pendant chloride into iodide **51** was attempted via Finckelstein conditions, however, even when utilising phase-transfer conditions in order to maintain a homogeneous flow regime the outcome was not satisfactory giving only low conversions. Alternatively direct amination of chloride **49** utilising high temperature flow conditions (140 °C) allowed the direct preparation of amine **50** in excellent yield. Flow processing using a short residence time (10 min) at the elevated temperature allowed for a good throughput; in addition, the handling of the volatile methylamine within the confines of the flow reactor simplifies the practical aspects of the transformation, however, extra precautions were required in order to address and remove any leftover methylamine that would pose a significant hazard during scaling up.

The final arylation of **50** was intended to be performed as a S_N_Ar reaction, however, insufficient deprotonation of the alcohol **50** under flow conditions (NaHMDS or BEMP instead of using a suspension of NaH as used in batch) required a modification to the planned approach. To this end a Mitsunobu protocol based on the orchestrated mixing of four reagent streams (**50**, **54** and reagents **52** and **53**) was developed and successfully applied to deliver fluoxetine (**46**) in high yield. Overall, this study is a good example detailing the intricacies faced when translating an initial batch synthesis into a sequence of flow steps for which several adaptations regarding choice of reagents and reaction conditions are mandatory in order to succeed.

**Scheme 9:**
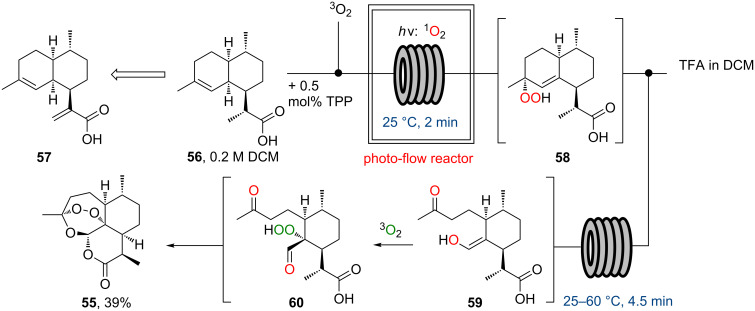
Flow synthesis of artemisinin (**55**).

The flow synthesis of the high profile antimalaria agent artemisinin (**55**) was reported by the Seeberger group in 2012 [61–62]. This intriguing approach represents one of the few examples where photochemistry has been employed in the synthesis of a pharmaceutical. For this endeavour dihydroartimisinic acid (**56**), an advanced building block that is available via chemoselective batch reduction of bioengineered artemisinic acid (**57**), was chosen as the starting point. The key transformations to yield artemisinin thus demanded a reaction cascade including a singlet oxygen mediated ene-reaction, a Hock cleavage of the resulting hydroperoxide **58** followed by oxidation with triplet oxygen and a final peracetalisation (Scheme 9).

Based on previous work by the Seeberger group and others [63–65] a simple flow photoreactor set-up comprising of a layer of FEP-polymer tubing wrapped around a cooled medium pressure mercury lamp was used to efficiently generate and react the singlet oxygen in the presence of tetraphenylporphyrin (TPP) as a photosensitizer. Upon exiting the photoreactor, the reaction stream was acidified by combining with a stream of TFA in order to enable the remaining reaction cascade to take place in a subsequent thermal reactor unit. After off-line purification by silica gel chromatography and crystallisation artemisinin was isolated in 39% yield equating to an extrapolated productivity of approximately 200 g per day.

**Scheme 10:**
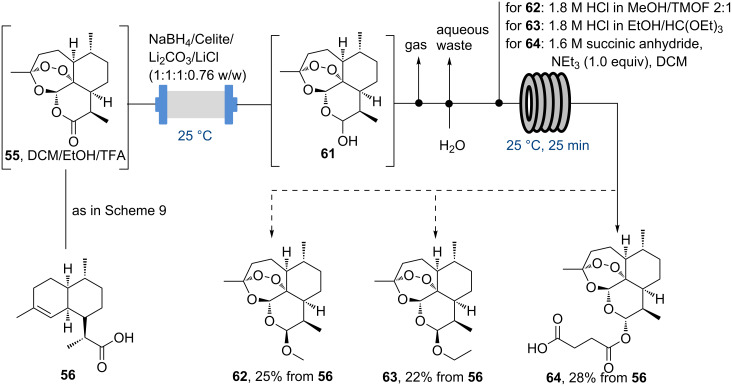
Telescoped flow synthesis of artemisinin (**55**) and derivatives (**62**–**64**).

More recently, Seeberger and McQuade reported on further improvements of this strategy enabled by the development of a NaBH_4_-based flow reduction procedure of artemisinin (**55**) to yield dihydroartemisinin (**61**) as well as in-line purifications and derivatisations to also generate several related malaria medications (i.e., β-artemether (**62**), β-artemotil (**63**) and α-artesunate (**64**)) in an efficiently telescoped manner (Scheme 10) [66–67].

As the authors mention, their work is related to an earlier study by researchers from the Universities of Warwick and Bath describing a continuous reduction protocol of artemisinin using LiBHEt_3_ in 2-Me-THF as a greener solvent [68]. Although this reductant is more expensive than NaBH_4_ this approach convinces through its simplicity and superior productivity (~1.6 kgh^−1^L^−1^).

Beside the use of photochemical processing towards the synthesis of artemisinin and its derivatives, this strategy has also been employed in the flow synthesis of a carprofen analogue [69] as well as in the regioselective bromination towards a rosuvastatin precursor [70] showcasing how continuous flow photochemistry is receiving a significant level of interest. This is not least because of the perceived green reagent concept of photons and the ability to overcome the inherent dilution problems encountered in batch. The ability to control residence times and hence decrease secondary transformations whilst using the small dimensions of the microreactor flow streams to enhance the photon flux has been claimed to increase productivity. However, it should be noted that many of the articles promoting the use of flow photochemistry do not currently adequately quantify or describe the systems in sufficient detail in order to fully justify such statements [65]. This is a general consideration but especially pertinent to the use of low power LED’s which are becoming increasingly popular. The calibration and quantification of the incident light from such devices is not normally evaluated or even commented upon in many of these studies hence reproducibility is therefore a major issue. Considering one of the main drivers of flow chemistry is an increase in reproducibility this seems a rather negative trend.

**Scheme 11:**
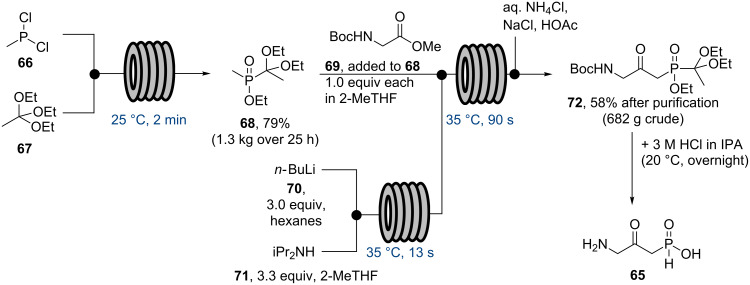
Flow approach towards AZD6906 (**65**).

In 2012 researchers from AstraZeneca (Sweden) reported upon a scale-up campaign for their gastroesophageal reflux inhibitor programme. Specifically, flow chemical synthesis was used to efficiently and reliably provide sufficient quantities of the target compound AZD6906 (**65**), which had been prepared previously in batch. From these earlier batch studies concerns had been raised regarding exothermic reaction profiles as well as product instability which needed to be addressed when moving to larger scale synthesis. Flow was identified as a potential way of circumventing these specific problems and so was extensively investigated. The developed flow route [71] started with the reaction of methyl dichlorophosphine (**66**) and triethyl orthoacetate (**67**), which in batch could only be performed under careful addition of the reagent and external cooling using dry ice/acetone. Pleasingly, a simple flow setup in which the two streams of neat reagents were mixed in a PTFE T-piece maintained at 25 °C was found effective in order to prepare the desired adduct **68** in high yield and quality showcasing the benefits of superior heat dissipation whilst also safely handling the toxic and pyrophoric methyl dichlorophosphine reagent (Scheme 11).

As the subsequent Claisen condensation step was also known to generate a considerable exotherm, a similar flow setup was used in order to allow the reaction heat to dissipate. The superiority of the heat transfer process even allowed this step to be performed on kilogram quantities of both starting materials (**68**, **69**) at a reactor temperature of 35 °C giving the desired product **72** within a residence time of only 90 seconds. Vital to the successful outcome was the efficient in situ generation of LDA from *n*-BuLi and diisopropylamine as well as the rapid quenching of the reaction mixture prior to collection of the crude product. Furthermore, flow processing allowed for the reaction of both substrates in a 1:1 ratio (rather than 2:1 as was required in batch) as the immediate quenching step prevented side reactions taking place under the strongly basic conditions. Having succeeded in safely preparing compound **72** on kilogram scale, the target compound **65** was then generated by global deprotection and subsequent recrystallisation where batch was reverted to as the conditions had been previously devised and worked well.

**Scheme 12:**
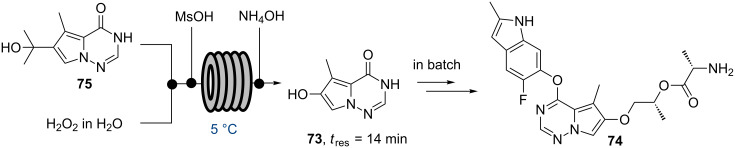
Pilot scale flow synthesis of key intermediate **73**.

As seen above, avoiding detrimental exotherms in scale up campaigns is a common reason for developing a continuous flow process. This approach is also demonstrated in the synthesis of the pyrrolotriazinone **73** via a exothermic oxidative rearrangement from **75**, a key intermediate towards brivanib alaninate (**74**) that was reported by researchers at BMS in 2014 (Scheme 12) [72].

**Scheme 13:**
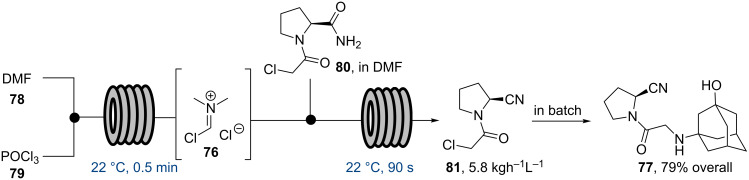
Semi-flow synthesis of vildagliptine (**77**).

Another application that undoubtedly benefits from performing scale up processes continuously concerns the generation and use of the Vilsmeier reagent (**76**). An early study by scientists at Roche (UK) demonstrated an approach in which Auto-MATE equipment combined with reaction simulation software was used to predict heat flow data for making and using Vilsmeier reagent at scale [73]. Using this information the formylation of 3,5-dimethoxyphenol was then performed at multi-kilo scale showing good agreement of the results with the devised simulations. More recently, scientists at Novartis (Switzerland) extended this study by developing a semi-continuous flow approach for the synthesis of the oral antidiabetic vildagliptine (**77**) using in situ generated Vilsmeier reagent (Scheme 13) [74].

Neat streams of DMF and POCl_3_ were mixed in a simple Teflon T-piece before entering a tubular reactor maintained at 22 °C (4.5 mL, *t*_res_ = 30 s). Upon exiting this reactor the crude stream of the Vilsmeier reagent **76** was combined with a stream of amide **80** in DMF that was prepared in situ in a batch reactor from proline amide and chloroacetyl chloride. The crude nitrile product **81** was then collected in a batch vessel and isolated in pure form after crystallisation and washing with *n*-heptane. Alkylation of **81** with the corresponding amino-adamantane derivate in the presence of excess K_2_CO_3_ following an existing batch protocol completed the synthesis of vildagliptine (**77**). Again, it was highlighted that the control of the exothermic Vilsmeier reagent formation and subsequent handling of this toxic and unstable intermediate was ideally suited to a continuous production and consumption in flow protocol.

### Gaseous reagents in flow

**Scheme 14:**
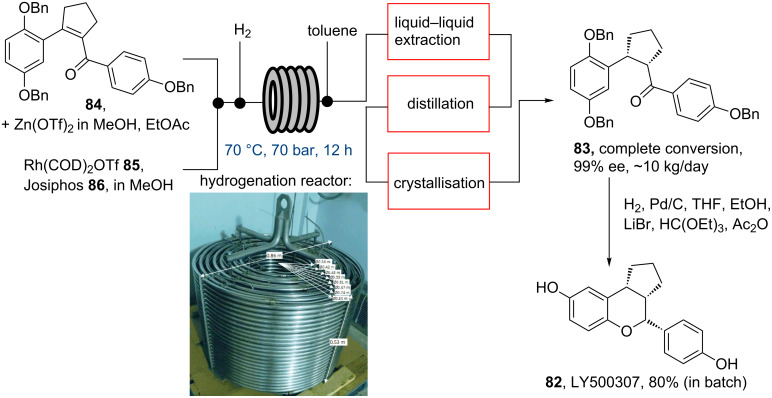
Pilot scale asymmetric flow hydrogenation towards **83**. Inserted photograph reprinted with permission from [75]. Copyright 2012 American Chemical Society.

Another example in which flow chemical synthesis was used as the key step in an industrial setting was reported by scientists from Eli Lilly (USA) in 2012. An asymmetric high-pressure hydrogenation towards LY500307 (**82**) [75] was demonstrated (Scheme 14). As this campaign aimed to produce the key intermediate **83** at pilot-scale, a flow-based asymmetric hydrogenation was chosen as an economically more viable option compared to establishing a high-pressure batch process.

As depicted in Scheme 14, solutions of the substrate **84** and a zinc triflate additive were combined with the rhodium precatalyst (**85**, 0.025 mol %, and Josiphos ligand **86**) before being mixed with hydrogen gas and entering a plug flow tubular reactor (volume 1.46 or 73 L, hydrogen pressure 70 bar, 70 °C, residence time 12 h). Several campaigns were run over periods of several days (e.g., campaign 1: 282 hours total cumulative reagent feed time) in order to evaluate this hydrogenation process. The process proved robust allowing reproducible and safe generation of the desired product in both high yield and enantiomeric excess. Additionally, semi-continuous liquid–liquid extraction, in-line distillation and product crystallisation were coupled to this hydrogenation step allowing for a total of 144 kg of the product **83** to be produced, purified and isolated using equipment that fits into existing laboratory fume hoods and hydrogenation bunkers. As the authors point out, this flow process not only delivered the hydrogenation product **83** with an improved safety profile at pilot-scale in a cost-effective manner, but moreover gave the same weekly throughput as a 400 L plant module operating in batch mode.

As the preceding examples clearly illustrate flow chemistry has quickly proven a viable means to assemble complex target molecules in a continuous and more modern fashion thus starting to satisfy claims regarding its advantageous nature compared to batch synthesis. Whilst some of these early examples can be seen as proof of concept studies, others have already demonstrated the application of further strategic elements including in-line purification and in-line analysis, both being crucial in order the achieve multistep flow synthesis. As the reader will see in the following part of this review, further advancements are geared towards more readily scaled processes and will also include the development of new devices allowing safe and efficient use of gaseous reagents as well as more effective ways of quickly transitioning between very low and very high temperatures that are key for streamlining modern flow synthesis routes.

**Figure 3:**
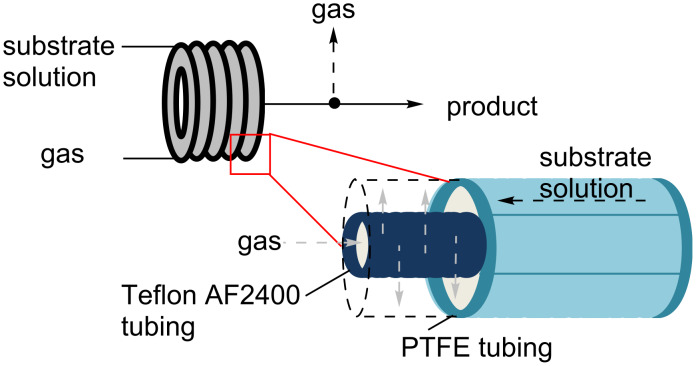
Schematic representation of the ‘tube-in-tube’ reactor.

Although the widely used H-Cube system had provided a popular solution for safe and convenient hydrogenation reactions at lab scale [76–79], the safe utilisation of other gaseous reagents at above ambient pressure was a relatively neglected area in flow chemistry for a long time. Only a few examples of flow hydrogenations and carbonylations had been reported [80–83]. The redevelopment and commercialisation of a laboratory based tube-in-tube reactor by the Ley group in 2009 changed the playing field and popularised the wider use of gases and volatile components. The design of the tube-in-tube system is based on a semipermeable Teflon AF2400 tubing (1 mm o.d., 0.8 mm i.d.) being housed within a wider PTFE tube (3.2 mm o.d., 1.6 mm i.d.; Figure 3). Depending on the intended application the gas can be fed either into the inner or the outer tube and upon pressurisation penetrates into the reagent stream where the desired reaction occurs. It has also been shown that an applied vacuum can enable the extraction of gaseous substances from a flow stream.

**Scheme 15:**
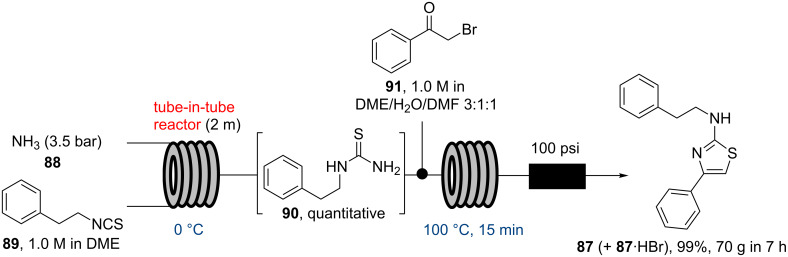
Flow synthesis of fanetizole (**87**) via tube-in-tube system.

This concept has since been studied in a variety of applications using for instance O_3_, CO, H_2_, CO_2_, O_2_, NH_3_ or syngas and has been reviewed very recently [84]. One noteworthy application of the tube-in-tube system by the Ley group in 2013 details the flow synthesis of the anti-inflammatory agent fanetizole (**87**) [85], in which ammonia gas was fed into the inner tube, whilst the outer tube contains a solution of phenethylisothiocyanate (**89**) in DME. The tube-in-tube system was placed onto the cooling unit of a Polar Bear Plus system maintained at 0 °C in order to generate the urea adduct **90** in quantitative yield (Scheme 15). In order to prepare the target compound this flow stream was then combined with an additional stream of bromoacetophenone (**91**) and passed through a heated tubular reactor unit (100 °C, 15 min) furnishing the 2-aminothiazole core of fanetizole (**87**). Due to preceding studies on the use of ammonia gas in this tube-in-tube system including in-line titrations only a minimal excess of gas (1.06 equivalents) was necessary to obtain complete conversion in the initial reaction subsequently allowing safe scale-up with a productivity of 70 g fanetizole (**87**) in 7 h.

**Scheme 16:**
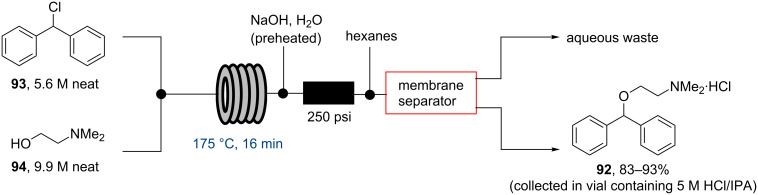
Flow synthesis of diphenhydramine^.^HCl (**92**).

In 2013 the Jamison group reported the flow synthesis of the important H_1_-antagonist diphenhydramine·HCl (**92**) showcasing the potential of modern flow chemistry to adhere to green chemistry principles (minimal use of organic solvents, atom economy etc.) [86]. The synthetic strategy relied on reacting chlorodiphenylmethane (**93**) with an excess of dimethylaminoethanol (**94**) via a nucleophilic substitution reaction (Scheme 16).

As both starting materials are liquid at ambient temperature the use of a solvent could be avoided allowing direct generation of the hydrochloride salt of **92** in a high temperature reactor (175 °C) with a residence time of 16 min. Conveniently at the same reaction temperature the product was produced as a molten paste (m.p. 168 °C) which enabled the continued processing of the crude product circumventing any clogging of the reactor by premature crystallisation. Analysis of the crude extrude product revealed the presence of minor impurities (<10%) even when stoichiometric amounts of **94** were used, consequently an in-line extraction process was developed. Additional streams of aqueous sodium hydroxide (3 M, preheated) and hexane were combined with the crude reaction product followed by passage through a membrane separator. The hexane layer was subsequently collected and treated with hydrochloric acid (5 M in IPA) leading to the precipitation of diphenhydramine hydrochloride (**92**) in high yield (~90%) and purity (~95%). Furthermore, options to further reduce waste generated during the purification sequence are presented by combining hot IPA with the crude flow stream leading to the isolation of the target compound (**92**·HCl) by direct crystallisation in the collection vessel (yield 71–84%, purity ~93%, productivity 2.42 g/h).

**Scheme 17:**
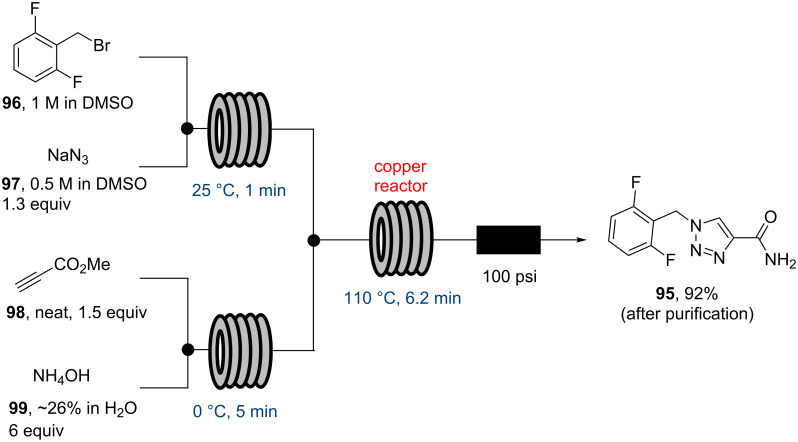
Flow synthesis of rufinamide (**95**).

More recently, the Jamison group also reported upon a short flow synthesis of the antiepileptic agent rufinamide (**95**) [87]. The 1,2,3-triazole ring was prepared via a dipolar cycloaddition between an in situ generated benzylic azide and propiolamide (also prepared in situ), which by maintaining a low inventory of the reactive intermediate reduced the safety concerns associated with the use of the azide. The choice of flow when handling hazardous materials like azides is a very frequently encountered driver and several publications detailing the associated benefits have emerged over the years [88–90]. Importantly in this study, a flow reactor consisting of copper tubing maintained at 110 °C (6.2 minutes residence time) was employed as this would release small amounts of copper salts catalysing the regioselective triazole formation. A cautionary note regarding the potential of generating copper azide within the reactor should be made here from a scale-up perspective as this was not explored in the paper. Overall, this small scale syringe pump based set-up enabled the preparation of rufinamide (**95**) within ~11 minutes processing time and with a productivity of ~0.22 g/h, although this does not take into account the time required for work-up and purification necessary to isolate the pure rufinamide (Scheme 17).

**Scheme 18:**
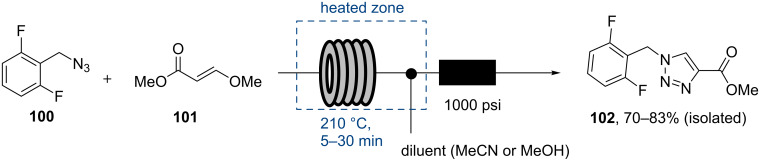
Large scale flow synthesis of rufinamide precursor **102**.

Although the above approach generates rufinamide (**95**) in a continuous fashion, a more convincing strategy towards rufinamide has been reported by the Hessel laboratory in 2013 [91]. Their route focused upon a dipolar cycloaddition between azide **100** and (*E*)-methyl 3-methoxyacrylate (**101**) to yield triazole **102** that was converted into rufinamide (**95**) (Scheme 18).

The benefits of using this alternative dipolarophile **100** are that it is not only considerably cheaper and less toxic than **98**, but it also delivers the desired 1,4-triazole regioisomer without the need for a metal catalyst that requires stringent purification afterwards. Due to the reduced reactivity of **100** the crucial cycloaddition step was conducted neat at elevated temperature (210 °C) yielding pure **102** within short residence times (5–30 minutes studied) on 20–200 mmol scale after crystallisation (70–83% yield).

**Scheme 19:**
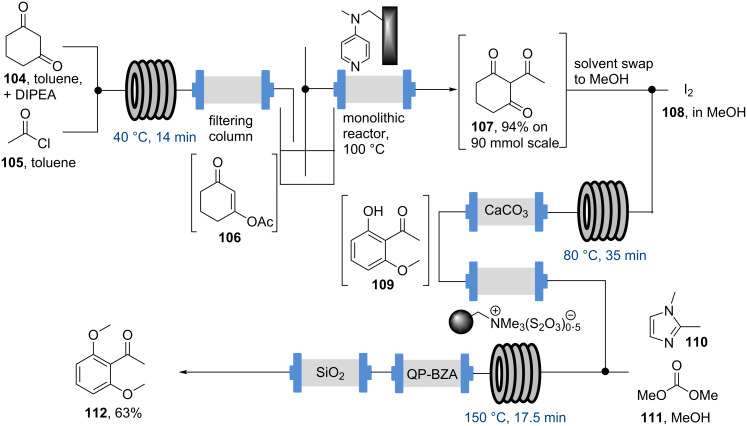
First stage in the flow synthesis of meclinertant (**103**).

As in the case of rufinamide (**95**), the choice of the flow reactor also plays a key role in the synthesis of meclinertant (SR48692, **103**), which is a potent probe for investigating neurotensin receptor-1 [92]. The flow synthesis of this challenging compound was reported in 2013 and aims to evaluate the benefits of flow chemistry in order to avoid shortcomings of previous batch synthesis efforts particularly in regard to scale up [93]. The investigation first involved the preparation of the key acetophenone starting material **112** which although commercially available was expensive and could be generated from 1,3-cyclohexadione (**104**). The sequence consisted of *O*-acetylation, a Steglich rearrangement, oxidation and a final methylation reaction. As the use of flow chemistry had already improved the *O*-acetylation during scale-up tests (130 mmol) by avoiding exotherms, it was anticipated that the subsequent Steglich rearrangement could be accomplished in flow using catalytic DMAP instead of stoichiometric AlCl_3_ as precedented (Scheme 19). This was eventually realised by preparing a monolithic flow reactor functionalised with DMAP that proved far superior to commercially available DMAP on resin. Employing the monolithic reactor cleanly catalysed the rearrangement step when a solution of **106** was passed through the reactor at elevated temperature (100 °C, 20 min residence time). The resulting triketone **107** was telescoped into an iodine mediated aromatisation, followed by high temperature mono-methylation using dimethyl carbonate/dimethylimidazole as a more benign alternative to methyl iodide at scale.

**Scheme 20:**
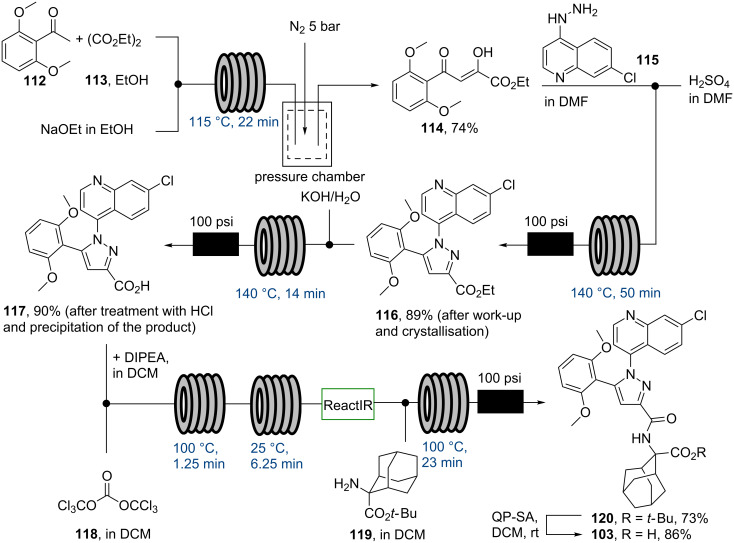
Completion of the flow synthesis of meclinertant (**103**).

The subsequent Claisen condensation step between ketone **112** and diethyl oxalate (**113**) was reportedly hampered by product precipitation and clogging problems, thus a pressure chamber was developed [94] that would act as a pressure regulator allowing this step to be scaled up in flow in order to provide **114** on multigram scale (134 g/h). A Knorr pyrazole formation between **114** and commercially available hydrazine **115** had previously been found difficult to scale up in batch (the yield dropped from 87% to 70%) and was thus translated into a high temperature flow protocol (140 °C) delivering the desired product **116** in 89% yield (Scheme 20). Ester hydrolysis and a triphosgene (**118**) mediated amide bond formation between acid **117** and adamantane-derived aminoester **119** [95] completed this flow synthesis. Meclinertant (**103**) was subsequently obtained after batch deprotection using polymer supported sulfonic acid. Overall, this study showcases how flow chemistry can be applied to gain benefits when faced with problems during mesoscale synthesis of a complex molecule. However, despite the successful completion of this campaign, it could be argued that the development time required for such a complex molecule in flow can be protracted; therefore both synthetic route and available enabling technologies should be carefully examined before embarking upon such an endeavour.

### New flow heating approaches

**Scheme 21:**
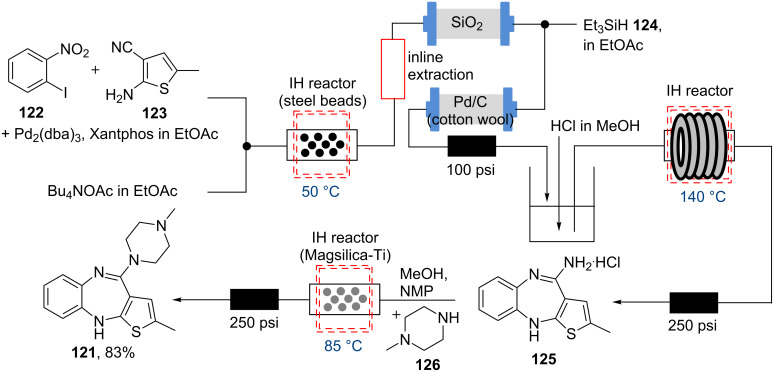
Flow synthesis of olanzapine (**121**) utilising inductive heating techniques.

One of the main advantages of flow chemistry is the safety and ease with which reactions can be performed continuously at elevated temperatures. With the exception of flow microwave constructs [96–101] all other reactor types rely on convective heat transfer. Although this is rapid for small reactor dimensions as the scale of the device increases the efficacy of the heating rapidly falls. The Kirschning group has introduced inductive heating (IH) as an energy stimulus for continuous flow synthesis [102–103]. In this scenario magnetic or conductive materials (metal beads, nanoparticles, etc.) are placed within a reactor cartridge exposed to an oscillating magnetic field of medium (15–25 kHz) or high frequency (780–850 kHz) leading to very rapid heating of reagent streams pumped through the reactor. A powerful application of this new concept was demonstrated in the flow synthesis of the atypical neurolepticum olanzapine (**121**) [104]. The synthesis begins with a Buchwald–Hartwig coupling between 2-iodonitrobenzene (**122**) and 2-aminothiophene **123** enabled by inductive heating (Scheme 21).

After in-line extraction and filtration through a silica packed cartridge, the resulting reaction stream was mixed with triethylsilane (**124**) and telescoped into a Pd-doped fixed bed reactor in order to affect smooth reduction of the nitro group. The output stream was then collected, and reintroduced to a flow reactor to be combined with a stream of dilute hydrochloric acid and passed through an inductively heated tubular reactor maintained at 140 °C to furnish benzodiazepine **125** in 88% yield after 30 h processing time. The flow synthesis of olanzapine (**121**) was completed by directing a mixture of benzodiazepine **125** and *N*-methylpiperazine (**126**) through a final inductively heated reactor containing Ti-doped Magsilica (85 °C, 83% after 15 h processing). Whilst this study did not aim to produce olanzapine at scale it aptly demonstrates the successful development and adaptation of inductive heating to the flow synthesis of this important pharmaceutical.

The Kirschning group (2013) also demonstrated a multi-step flow synthesis of the antidepressant amitriptyline (**127**) [105]. They developed a sequence harnessing reactions at several different temperature regimes allowing processing of low temperature lithiation and carboxylation reactions, ambient temperature Grignard addition and the high temperature elimination of water.

**Scheme 22:**
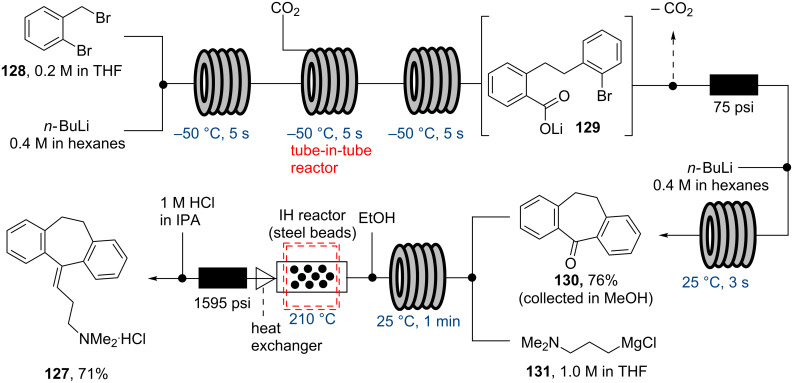
Flow synthesis of amitriptyline·HCl (**127**).

In the process solutions of 2-bromobenzylbromide (**128**) and *n*-BuLi are delivered into a small tubular flow reactor maintained at −50 °C in order to perform a Wurtz-type coupling. The resultant aryllithium intermediate passes into a tube-in-tube reactor, where carboxylation takes place furnishing the lithium carboxylate **129**. Excess carbon dioxide is subsequently removed using a degassing tube before reacting species **129** with a further stream of *n*-BuLi to induce cyclisation to dibenzosuberone (**130**) in a short total residence time of 33 seconds (Scheme 22). Finally, the stream of **130** was combined with 3-(dimethylamino)propylmagnesium chloride (**131**) to affect a Grignard addition at ambient temperature followed by passage through an inductively heated reactor (210 °C, 810 kHz, 36 seconds residence time) which under the acidic conditions promotes dehydration. The product is isolated as the in situ formed hydrochloride salt of amitriptyline (**127**).

### Solution delivery

As the previous examples have demonstrated, the development of an efficient flow process is often the result of designing and implementing a new concept or piece of equipment that is better suited to performing an otherwise challenging task. One aspect of continuous flow synthesis for which little progress was made for a long time concerned the way in which reagents streams were delivered into the reactors. In much of the early flow chemistry work delivery of liquid streams was achieved using simple syringe pumps. Unfortunately syringe pump applications are significantly limited by relatively low working pressures and often needed manual intervention when recharging the syringe which precluded a fully continuous and automated process. Alternatively the use of piston or rotary pumps (i.e., HPLC pumps) could be employed but these also have drawbacks being often characterised by inaccurate flow rates or fouling over prolonged periods of use due to their direct interactions with the chemicals being pumped (for continuous flow applications not using a sample loop). In addition both of these pumping solutions require homogeneous solutions where particulates or precipitates (slurries) are extremely detrimental. These shortcomings obviously impact the performance of flow reactors when attempting reaction scale-up, especially when precise and consistent reagent delivery is crucial.

**Scheme 23:**
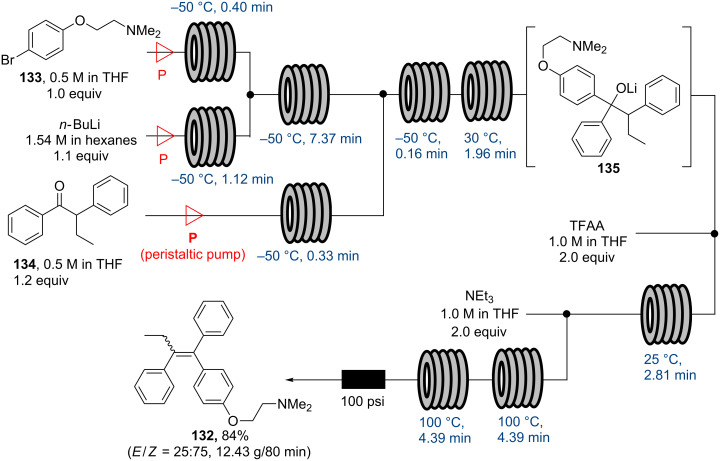
Flow synthesis of *E*/*Z*-tamoxifen (**132**) using peristaltic pumping modules.

In order to address these issues flow equipment utilising adapted peristaltic pumps have been developed and applied to several mesoscale syntheses utilising common organometallic reagents (i.e., *n*-BuLi, Grignard reagents, DIBAL-H) [106]. The pump design uses specific fluorinated polymers for the feed tubing that is placed on the rotor of a modified peristaltic pump resulting in a smooth and consistent delivery of a solution that can be drawn directly out of the supplier’s reagent bottle. A first application of a commercial system was reported by the Ley group with their continuous synthesis of the important anticancer agent tamoxifen (**132**) in 2013 [106]. The synthesis starts with a halogen–lithium exchange reaction between arylbromide **133** and *n*-BuLi at −50 °C and the subsequent addition of the formed aryllithium species to ketone **134** at the same temperature (Scheme 23). After exiting the cold reactor zone, this stream passes through a coil maintained at 30 °C in order to ensure the complete consumption of the ketone **134**.

The resulting solution of lithium alkoxide **135** is combined with a further stream containing trifluoroacetic anhydride (TFAA) before being mixed with a stream of triethylamine in order to promote the elimination of the activated tertiary alcohol. A good isolated yield of (*E/Z*)-tamoxifen (**132**) (84%, *E*/*Z* ratio 25:75) was achieved after trituration with hot hexanes. As this report states, the peristaltic pumping module used in this synthesis permitted a production of almost 13 g of (*E*/*Z*)-tamoxifen over a period of only 80 minutes, providing sufficient material for one patient’s treatment for 900 days.

As this example demonstrates, flow chemistry can be used as a means to facilitate the direct synthesis of a supply of pharmaceuticals from a small dedicated reactor. This enables the quick and easy relocation of manufacturing to permit medications to be made bespoke at the site of requirement or in future applications on demand as required by the patient or prescriber.

**Figure 4:**
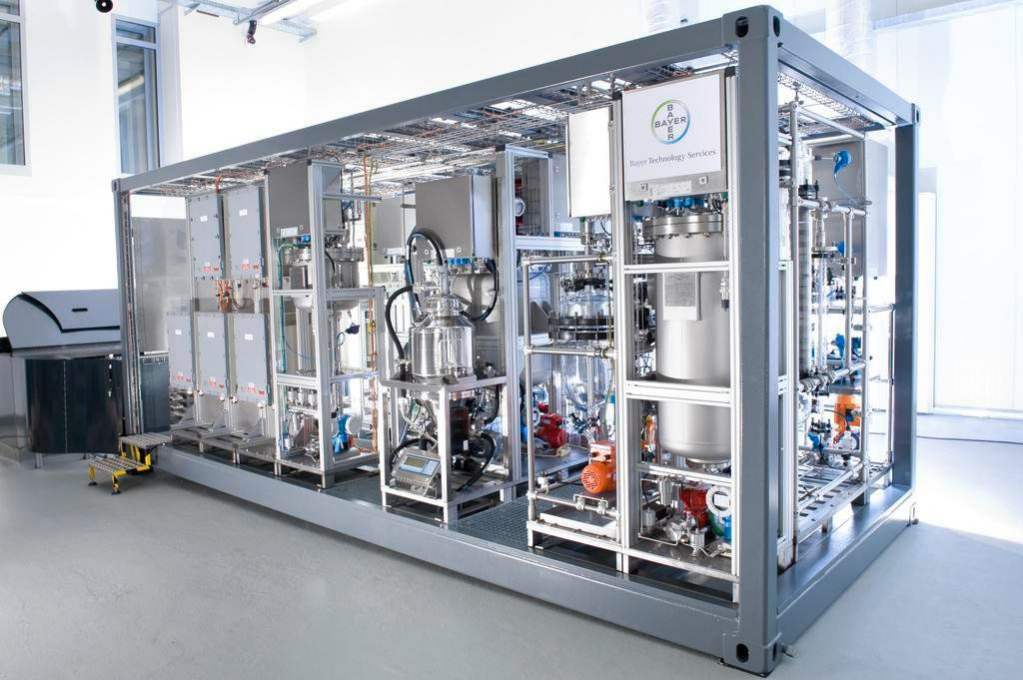
Container sized portable mini factory (photograph credit: INVITE GmbH, Leverkusen Germany).

It is also worth highlighting here two European initiatives in this regrad, namely CoPIRIDE [107] and the F³ Factory which have both focused on developing new technologies, processes and manufacturing concepts towards the “chemical plant of the future” [108]. One of the specific goals of the CoPIRIDE project was the design of a small footprint modular chemical plant to be embedded in a standard EU 20-foot ISO norm container (3 × 12 m storage container). Extensive use of flow chemistry and microreactor technologies were used to create a 'plug-and-play', container-based production facility (Figure 4). The challenge was to create a flexible facility that could be easily reconfigured to generate multiple chemical outputs as required. This shift towards greater versatility and a smaller environmental footprint also provide for the easy and rapid redeployment of the unit at a new geographical location making it more capable of adapting to market trends and changing manufacturing demands. Several working units have been assembled and successfully used for a range of chemistries including hydroformulations, biodiesel and acrylic acid production and large scale polymerisation reactions [109].

### Flow manufacturing

Whereas the previous applications have demonstrated how flow chemistry can enable the rapid preparation of several pharmaceuticals by focusing on the synthetic effort, the final examples in this review showcase how flow synthesis can be linked to in-line assaying of new molecules as well as the continuous manufacture and formulation of drug compounds.

In 2013 the Ley group disclosed a study detailing the flow synthesis of a library of GABA_A_ agonists which was linked to in-line frontal affinity chromatography (FAC) in order to directly generate binding affinity data for these new entities towards human serum albumin (HSA), a highly abundant protein in human blood plasma [110].

**Scheme 24:**
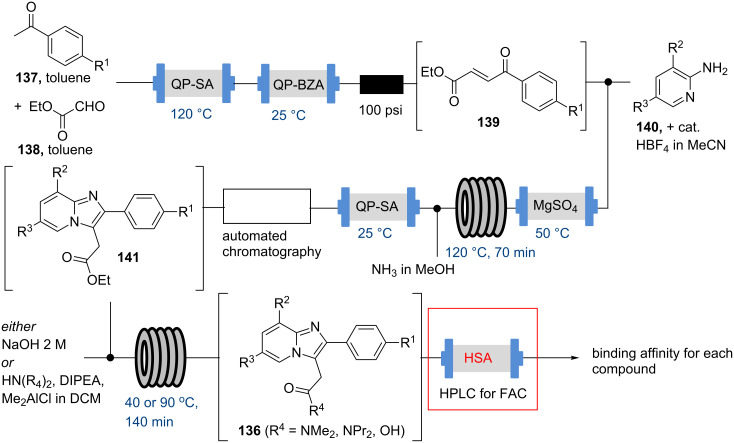
Flow synthesis of imidazo[1,2-*a*]pyridines **136** linked to frontal affinity chromatography (FAC).

The synthesis of a small collection of imidazo[1,2-*a*]pyridine derivatives was realised through the application of different scavenger resins for in-line purification as well as a number of liquid handlers to orchestrate the library synthesis effort (Scheme 24). Using this semi-automated process a small collection of 22 imidazo[1,2-*a*]pyridines **136** was prepared within four working days. The synthetic route consisted of an aldol condensation between various acetophenones **137** and ethyl glyoxylate (**138**). This was followed by an HBF_4_-catalysed cyclocondensation of the resulting Michael acceptor **139** with various 2-aminopyridines **140** and subsequent derivatisation of the ester group into the corresponding acid or amide moiety.

**Figure 5:**
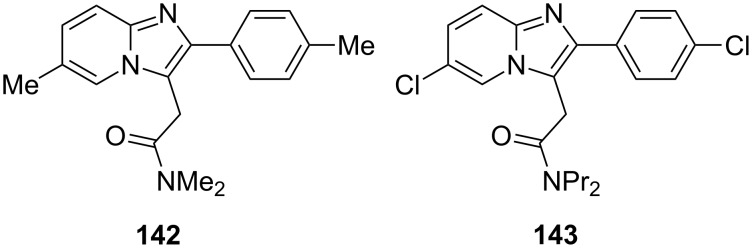
Structures of zolpidem (**142**) and alpidem (**143**).

In order to directly perform the FAC analysis on these structures an HPLC column (15 µL volume) was filled with commercially available HSA protein and connected to a HPLC system. After establishing the void volume of this column, two different literature known marker compounds (diclofenac sodium and isoniazid) were used in order to calibrate the system based on their retention time which could be directly correlated to the protein binding affinity. Furthermore, as the compound library contained zolpidem (**142**) and alpidem (**143**) (Figure 5), two FDA approved drugs for which affinity data were already literature reported the authors were able to validate their method by matching their affinity data.

Using this setup, solutions of the remaining imidazo[1,4-*a*]pyridines (600 µL, 67.5 µM) were passed through the binding assay column allowing quick determinations of their HSA binding affinity. This proof of concept study therefore marks one of the first published reports where flow chemical synthesis is combined with direct biological evaluation of new structures thus linking chemistry with biology using standard flow equipment.

**Scheme 25:**
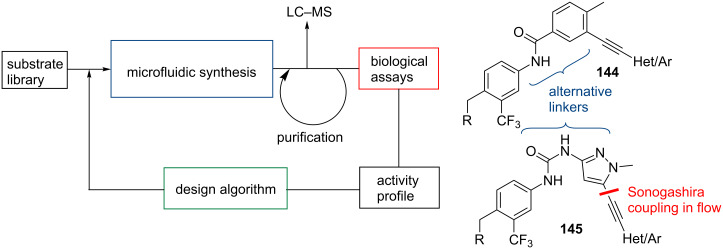
Synthesis and screening loops in the discovery of new Abl kinase inhibitors.

A second application demonstrating the power of this paradigm shift towards improving the hit-to-lead and lead optimisation was published in 2013 by researchers at Cyclofluidics, a company dedicated to the acceleration of preclinical discovery processes [111]. In this work a platform capable of designing a virtual chemical space was presented that further integrates the synthesis, purification and screening of the newly designed entities. Analogue optimisation was accomplished by running several microfluidic synthesis-screening loops that establish key SAR data. This approach was exemplified by synthesising a small library of Abl kinase inhibitors with the synthesis aspect focusing on the Sonogashira coupling between heterocyclic alkynes (hinge binder motif) and a selection of aryl iodides and bromides (DFG-binder motif) based on the common benzamide scaffold of ponatinib (**144**, R = *N*-methyl piperazine, Het = imidazopyridazine) and related pyrazole-ureas (**145**) (Scheme 25).

As depicted schematically in Scheme 25 the development cycle commences with the microfluidic synthesis of a new analogue followed by its in-line analysis (LC–MS) and purification (by passage through a silica cartridge). The clean compound is then assayed allowing the resulting activity profile to be fed into a design algorithm which determines which compound to next prepare and test. Using this repeating loop approach led the cyclofluidics scientists to the discovery of the pyrazole-urea motif **145** as a potential replacement of the more common benzamide systems **144**.

**Figure 6:**
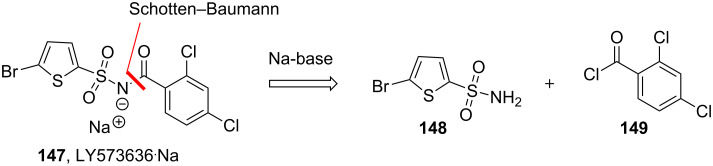
Schotten–Baumann approach towards LY573636**^.^**Na (**147**).

In 2014 researchers from Eli Lilly (US) disclosed a detailed study regarding the synthesis of LY2886721 (**146**), a then promising inhibitor of beta-amyloid cleaving enzyme (BACE). Their work focussed on evaluating flow techniques for the key amide bond forming step under modified Schotten–Baumann conditions [112]. This work was related to earlier studies at Eli Lilly detailing a continuous synthesis of the anticancer agent LY573636^.^Na (**147**, Figure 6) that also used a Schotten–Baumann reaction as key step [113]. The driving force in the development of a continuous process was in both cases to minimise exposure of individuals to hazardous materials by means of fewer unit operations, and more importantly the development of the concept of ‘tech transfer by truck’ meaning that once established, a continuous process could be easily replicated at a different location without need for major investments.

**Scheme 26:**
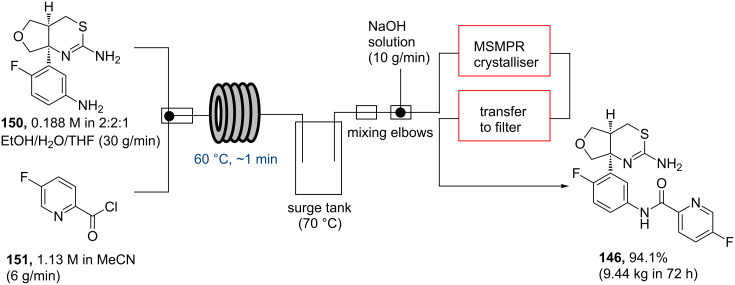
Pilot scale flow synthesis of LY2886721 (**146**).

As the effort to prepare LY2886721 targeted a pilot-scale process (up to 10 kg/72 h) high concentrations of diamine **150** and acid chloride **151** had to be successfully handled. The amide formation was conducted in a plug flow reactor followed directly by reactive crystallisation in a mixed suspension, mixed product removal (MSMPR) crystalliser (Scheme 26).

Because of the high concentrations and potential for solid formation peristaltic pumps were used to direct solutions of diamine **150** (in EtOH/water/THF 2:2:1) and acid chloride **151** (prepared in situ with oxalyl chloride in MeCN/cat. DMF) via a static mixer into a plug flow reactor (60 °C, up to 60 seconds residence time). The desired amide product **146** (as its hydrochloride) was rapidly formed in both high yield (>99%) and purity (>98%). Importantly, maintaining a temperature of 60 °C ensured that reactor fouling by precipitation did not occur despite the supersaturation of the product stream. The feed stream of **146** (hydrochloride) was passed into a surge tank (70 °C) and was subsequently directed into mixing elbows where it was combined with a stream of aqueous NaOH leading to the formation of the free-based **146** that would subsequently crystallise in the crystalliser (30 min residence time). This also required adhering to high specifications regarding crystal size and morphology. The sequence could be performed at scale and in an improved throughput of 139 g/h (vs 33 g/h as achieved in batch) yielding 9.44 kg of **146** over 72 h (yield of 94.1%). Overall, the success of this flow campaign was attributed to the improved reactive crystallisation as well as the relatively low process and equipment costs compared to earlier batch efforts.

**Scheme 27:**
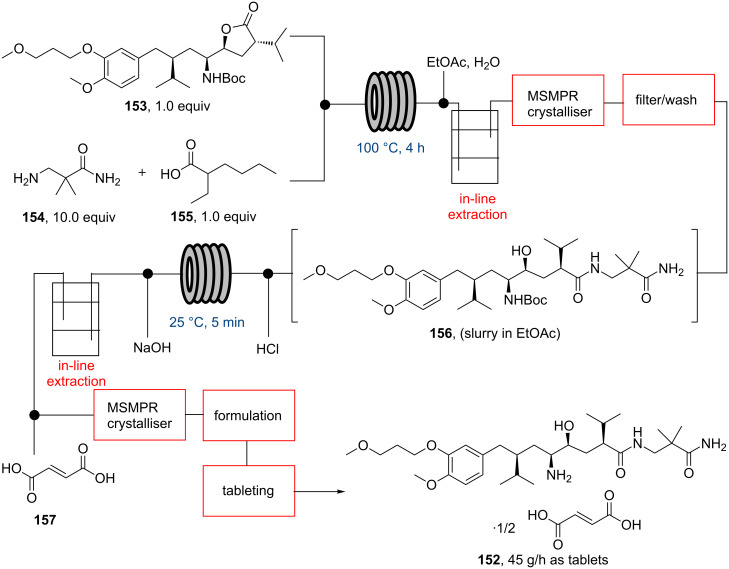
Continuous flow manufacture of alikiren hemifumarate **152**.

A final application was recently reported by a research team at MIT detailing a continuous flow process towards aliskiren hemifumarate (**152**), including synthesis, purification, formulation and tableting, thus demonstrating the continuous manufacture of a pharmaceutical [114]. This achievement was enabled by constructing a compact plant module (2.4 m × 7.3 m^2^) that can produce aliskiren (**152**) at a rate of 45 g/h equating to 2.7 million tablets per year. The synthetic sequence starts with the lactone ring opening of the very advanced intermediate **153** using amine **154** (10 equiv) and additive **155** (1 equiv) in a tubular reactor (100 °C, 4 h residence time) to furnish amide **156**. Importantly, as **156** was at this stage melted, no reaction solvent was required for this step (Scheme 27).

The exiting, hot melt stream was mixed combined with EtOAc and water to solubilise and extract the desired product into the organic layer. The organic phase was directed into a mixed suspension, mixed product removal (MSMPR) crystalliser where upon cooling and addition of heptane as an anti-solvent a slurry formed. After additional processing (washing/filtration) the amide slurry of **156** was telescoped into a further sequence furnishing aliskiren fumarate. This involved Boc deprotection, quenching, in-line extraction and final salt formation. The continuous formulation process also requires addition of an excipient (SiO_2_) prior to drying, which results in the generation of a solid cake that after grinding provides a tractable powder of **152** on SiO_2_. This material is mixed with 6000 Da PEG (35:65 mass ratio) and continuously fed into a heated extruder unit in order to mix and melt the components prior to tableting. Importantly, the tablets prepared successfully passed various quality control tests (visual appearance, size and dosage) and as residual impurities and solvents were found within specifications could be released as final formulated drugs.

Overall this application of continuous drug manufacture highlights the standing within the field by showcasing how a final dosage form of a pharmaceutical can be produced in a highly automated and continuous fashion by linking chemical synthesis and purification to direct formulation and final tableting. It still however remains to be demonstrated that a more comprehensive and fully integrated continuous synthesis and tableting sequence can be achieved. Although this work is an impressive achievement it should be acknowledged that the preparation involves only very limited and trivial chemistry. However, we have in the preceding parts of this review highlighted many impressive achievements demonstrating complex synthesis so all the individual components required to perform the unification have now been conducted. It will therefore only be a short time until more elaborate and convincing examples of end-to-end manufacturing are reported.

## Conclusion

As this review has clearly demonstrated, flow chemistry has matured from an innovative synthesis concept for improving chemical synthesis to a powerful and widely applicable tool box enabling the efficient multistep synthesis of numerous active pharmaceutical ingredients. Whilst the original developments came mainly from academic proof of concept studies the rapid uptake and disclosure of flow syntheses has now generated sufficient knowledge and equipment to execute any conceivable flow synthesis. Furthermore, this has inspired considerable progress in the linking of continuous synthesis to in-line purification, biological assaying, and indeed formulation of medications. At this point it remains to be seen as to whether continuous synthesis and manufacture of pharmaceuticals will be applied primarily to small volume drugs and personalised medicines, or if its benefits regarding safety, scale-up and automation will render continuous processing a key element across more higher volume products. Current estimates suggest a general increase in industrial applications of continuous manufacture of pharmaceuticals from 5% to 30% over the next few years. Various pharma corporations as well as regulatory authorities (FDA etc.) have fully advocated the use of continuous manufacture. Nevertheless, a number of bottlenecks still remain to be addressed in order to allow the community to fully appreciate and exploit the true value of continuous synthesis and manufacture. For one, it seems that there is still a significant gap between many flow approaches developed by academic groups and those needed to solve problems in industrial campaigns, however, exchange of experience by specific case studies is starting to bridge these discrepancies. Furthermore, with the commercialisation (and eventually reduced cost) of various modular flow reactors one can expect a further increase in flow-based applications. This trend might also be backed by the changing mind-set of the practitioner becoming more accustomed and confident in building and operating different flow reactors rather than relying on traditional batch based lab equipment. Crucial to this trend will be the training of students in flow chemistry by academics, which currently is clearly lagging behind expectation and demand. For this reason universities should be encouraged to develop lecture courses and practical classes to provide training in flow based chemical synthesis at undergraduate and postgraduate student level. If these adjustments can be made within the next few years, we can expect a continuing advancement of the field and the continuous manufacture of pharmaceuticals should become a common practice rather than a novel exception.
